# Isolated Human Pulmonary Artery Structure and Function Pre‐ and Post‐Cardiopulmonary Bypass Surgery

**DOI:** 10.1161/JAHA.115.002822

**Published:** 2016-02-23

**Authors:** Kim A. Dora, Christopher P. Stanley, Emad Al Jaaly, Francesca Fiorentino, Raimondo Ascione, Barnaby C. Reeves, Gianni D. Angelini

**Affiliations:** ^1^ Department of PharmacologyUniversity of OxfordUnited Kingdom; ^2^ Department of Cardiothoracic SurgeryHammersmith HospitalImperial College LondonLondonUnited Kingdom; ^3^ Bristol Heart InstituteBristol Royal InfirmaryUniversity of BristolUnited Kingdom

**Keywords:** bradykinin, cardiopulmonary bypass, lung, thromboxane, vasoconstriction, vasodilation, Cardiovascular Surgery, Contractile function, Pulmonary Biology, Coronary Artery Disease

## Abstract

**Background:**

Pulmonary dysfunction is a known complication after cardiac surgery using cardiopulmonary bypass, ranging from subclinical functional changes to prolonged postoperative ventilation, acute lung injury, and acute respiratory distress syndrome. Whether human pulmonary arterial function is compromised is unknown. The aim of the present study was to compare the structure and function of isolated and cannulated human pulmonary arteries obtained from lung biopsies after the chest was opened (pre–cardiopulmonary bypass) to those obtained at the end of cardiopulmonary bypass (post–cardiopulmonary bypass) from patients undergoing coronary artery bypass graft surgery.

**Methods and Results:**

Pre‐ and post–cardiopulmonary bypass lung biopsies were received from 12 patients undergoing elective surgery. Intralobular small arteries were dissected, cannulated, pressurized, and imaged using confocal microscopy. Functionally, the thromboxane mimetic U46619 produced concentration‐dependent vasoconstriction in 100% and 75% of pre‐ and post–cardiopulmonary bypass arteries, respectively. The endothelium‐dependent agonist bradykinin stimulated vasodilation in 45% and 33% of arteries pre‐ and post–cardiopulmonary bypass, respectively. Structurally, in most arteries smooth muscle cells aligned circumferentially; live cell viability revealed that although 100% of smooth muscle and 90% of endothelial cells from pre–cardiopulmonary bypass biopsies had intact membranes and were considered viable, only 60% and 58%, respectively, were viable from post–cardiopulmonary bypass biopsies.

**Conclusions:**

We successfully investigated isolated pulmonary artery structure and function in fresh lung biopsies from patients undergoing heart surgery. Pulmonary artery contractile tone and endothelium‐dependent dilation were significantly reduced in post–cardiopulmonary bypass biopsies. The decreased functional responses were associated with reduced cell viability.

**Clinical Trial Registration:**

URL: http://www.isrctn.com/ISRCTN34428459. Unique identifier: ISRCTN 34428459.

## Introduction

The pulmonary circulation receives the total cardiac output; yet in humans the characterization of pulmonary arteries is sparse. Most research into the structure of pulmonary arteries has been carried out using animal models or cast models from humans^1^ with rare studies of arterial wall thickness and morphology in sectioned tissue samples biopsied from patients.^2^, ^3^

In addition to the paucity of studies looking at the structure of human pulmonary arteries, there is no report in the literature on the physiological behavior of these arteries. An understanding of structure and function is important in order to characterize dysfunction and to investigate potential interventions to remedy derangements. A relevant context is cardiac surgery with cardiopulmonary bypass (CPB) where respiratory distress or dysfunction is one of the most common complications, affecting up to 25% of patients.^4^ Presumed causative factors include inflammation, prolonged lung collapse, pulmonary ischemia and related reperfusion injury, blood contact with the surface of the heart–lung machine, endotoxemia, surgical trauma, blood loss, and transfusion.^5^, ^6^ These factors have been associated with in‐hospital mortality, morbidity, and increased hospital costs.^7^, ^8^, ^9^ Current strategies to reduce the respiratory dysfunction/edema associated with cardiac surgery have been the following: technical modifications of CPB, hemodilution, postoperative administration of steroids, and a range of lung ventilation protocols.^10^ However, these modifications have had moderate or short‐lived therapeutic success. A better understanding of the circulatory changes responsible for the lung dysfunction associated with cardiac surgery with CPB may help devise strategies aimed at reducing postoperative pulmonary complications, but to date this has not been possible due to a lack of fresh lung tissue.

We took advantage of a randomized trial to obtain lung biopsies for studies of structure and function. The aims of the present study were 2‐fold: first, to demonstrate proof‐of‐principle that structural and functional assessments of human pulmonary arteries from lung biopsies are possible; and, second, to compare structural and functional assessments of human pulmonary arteries before and after CPB in patients undergoing coronary artery bypass graft (CABG).

## Methods

Patients undergoing CABG in the department of cardiothoracic surgery at the Hammersmith Hospital were recruited, after giving written informed consent, to a randomized controlled clinical trial comparing low‐frequency ventilation during CPB to standard care; the trial is registered as ISCTRN 34428459. Biopsy samples for a subgroup were transported to Oxford under a material transfer agreement. The study completed recruitment and follow‐up in June 2014. A favorable Research ethics opinion approval was granted by the Camden & Islington NRES Research Ethics Committee London (reference 12/LO/0458) in April 2012 and amended in April 2013 to allow the use of some biopsy tissue for this substudy. This research complies with the Helsinki Declaration.

### Study Participants

Patients aged ≥40 and <85 years and having elective or urgent CABG with CPB and cold blood cardioplegic arrest for CABG were eligible. Exclusion criteria were the following: left ventricular ejection fraction ≤30%, previous pulmonary embolism requiring warfarin for ≥3 months, previous cardiac surgery, NYHA class IV, cardiogenic shock, chronic renal failure requiring dialysis, treatment with corticosteroid or immunosuppressive drug, severe chronic obstructive pulmonary disease, lung pathology, previous radiotherapy, or body mass index >35.

### Surgical, Anesthetic, and Cardiopulmonary Techniques

Operations were carried out following standard protocols for the Hammersmith Hospital. Anesthetic, cardiopulmonary, and surgical techniques were as previously reported.^6^, ^11^ Briefly, after premedication with temazepam, anesthesia was induced with a combination of propofol and remifentanil; muscle relaxation was achieved using vecuronium. Anesthesia was maintained by infusion of propofol and remifentanil (5 mg remifentanil to 1 g propofol) to keep the entropy value of the processed electroencephalogram below 55. After intubation, all participants were ventilated mechanically until starting CPB. Mechanical ventilation used volume control at the following settings: tidal volume of 6 to 8 mL·kg^−1^, I:E ratio of 1:2, positive end‐expiratory pressure of 5 cm H_2_O, FiO_2_ of 0.5, and ventilatory rate as required to keep the PaCO_2_ between 4.5 and 5.5 kPa. CPB was conducted with systemic temperature between 32°C and 35°C. Cardioplegic arrest was achieved with antegrade cold blood cardioplegia.

Two lung biopsies were taken from each patient using the LigaSure Impact^™^ instrument (LF4318) connected to a ForceTriad^™^ energy platform (Covidien, Boulder, CO). The system combines TissueFect^™^ sensing technology with hand activation and an integrated cutting mechanism to achieve multifunctional tissue sealing. The pre‐CPB biopsy was taken from the left upper lobe after sternotomy and the post‐CPB biopsy was taken from the left lower lobe prior to weaning from CPB. Samples were placed in ice‐cold Ca^2+^‐free MOPS buffer solution (mmol/L: NaCl 145, KCl 4.7, MgSO_4_·7H_2_O 1.17, MOPS 2.0, NaH_2_PO_4_·H_2_O 1.20, glucose 5.0, pyruvate 2.0, EDTA 0.02, and NaOH 2.75 with pH adjusted to 7.40±0.02), transferred from surgery to the laboratory on ice, and dissection commenced within 2 to 4 hours. Once in the laboratory, samples were placed in Ca^2+^‐containing MOPS (composition as above with the addition of 2.0 mmol/L CaCl_2_·2H_2_O) and an intralobular artery was dissected free from surrounding tissue. The artery was cannulated onto glass micropipettes, heated to 36.5±0.4°C, pressurized to 40 mm Hg, and left to equilibrate for 30 minutes in a 2 mL chamber (RC‐27 chamber, PH‐6 platform; Warner Instruments, Hamden, CT), as previously described using other tissue.^12^

### Confocal Microscopy Studies

In all studies, arteries were visualized using Olympus linescan confocal microscopes (FV500 and FV1000). When studying function, arteries were imaged with transmitted light using a ×10 Olympus objective and recorded using Fluoview software (Olympus, Tokyo, Japan) at 1 Hz. The structure of arteries was studied with imaging using a ×40 (1.15 NA, 0.25 mm WD) water immersion Olympus objective, obtaining *z*‐stacks through the arterial wall in 0.5 or 1 μm steps to observe each cell type, in turn. Fluorescent dyes were used to stain cells with calcein (excitation 488 nm), and nuclei with Hoechst 33342 (excitation 405 nm) and propidium iodide (PI, excitation 543 nm), and postfixation, smooth muscle actin with phalloidin (excitation 543 nm), nuclei with DAPI (excitation 405 nm), and elastin with Alexa Fluor^®^ 633 hydrazide (AF‐633, excitation 633 nm).

### Functional Studies

Each artery was tested for leaks, assessed as an ability to hold diameter to >90% of original for a minute during a sealed pressure test. Small side‐branches were common and detected by rapid deflation of arteries during this test, and these arteries were therefore not used for experiments. On occasion, no arteries within a biopsy passed this leak test, causing the number of samples with data to be less than the maximum of 12, with incomplete pairing between pre‐ and post‐CPB biopsies for some patients.

To test contractile function of the arteries, concentration response curves were determined by varying the concentration of the thromboxane mimetic U46619 from 0.03 to 3 μmol/L. From a state of submaximal contraction to U46619, endothelial function (dilation of the arteries) was assessed using a single concentration of bradykinin (1 μmol/L). Finally, maximum contraction was measured at the end of the experiment using a combination of 3 μmol/L U46619 and 45 mmol/L KCl.

### Structural Studies

Studies of smooth muscle cell (SMC) orientation and density were carried out by fixing cannulated arteries with 2% (wt/vol) paraformaldehyde for 10 minutes at 36.5±0.4°C, washing with PBS, then incubating arteries overnight at 4°C with phalloidin‐TRITC (6.4 μmol/L); nuclei were stained with DAPI (1.5 μmol/L). The elastin present throughout the arterial wall was visualized with AF‐633 hydrazide (250 nmol/L), and the integrity of inter‐endothelial cell (EC) tight junctions assessed by immunohistochemistry (mouse monoclonal anti‐human ZO‐1, aas 334‐634, 1:200, Life Technologies 339100; Alexa Fluor^®^ 488 chicken anti‐mouse IgG 1:200, Life Technologies A‐21200) using established methods.^12^

Once it was clear that cell orientation and density per se did not relate to cell function, an additional protocol was applied at the completion of functional experiments, in unfixed tissue. Live cells were classified according to their ability to uptake and de‐esterify the fluorescent dye calcein AM, and classification was confirmed by comparing staining with cell permeant and impermeant nuclear dyes. This live cell staining analysis of SMCs and ECs was carried out by perfusing the artery lumen with calcein AM (1 μmol/L), Hoechst 33342 (Hoechst, cell‐permeant nuclear dye, stains live and dead cells, 16 μmol/L), together with PI (cell‐impermeant nuclear dye, stains dead cells, 10 μmol/L) at 8 μL/min for 30 minutes using a Beehive^®^ syringe pump system. Cells were classified as live (calcein in cytoplasm; nuclei stained with Hoechst but not PI) or dead (nuclei stained with both Hoechst and PI; no calcein); live cells were expressed as a percentage of all cells. The elastin was not specifically stained in this protocol, but the autofluorescence of elastin was observed.

### Materials

NaCl, KCl, and d‐glucose were purchased from Fisher Scientific (Loughborough, UK); MOPS, EDTA, CaCl_2_·2H_2_O, pyruvate, PBS sachets, Tween20, and bovine serum albumin were purchased from Sigma (Poole, UK); and MgSO_4_·7H_2_O, NaOH, and NaH_2_PO_4_·H_2_O were purchased from VWR (Leicestershire, UK). Bradykinin and U46619 were purchased from Tocris (Bristol, UK). The structural dyes Hoechst (H3570), PI (P1304MP), DAPI (D3571), and AF‐633 (A30634) were purchased from ThermoFisher Scientific (Paisley, UK) and phalloidin‐TRITC from Sigma‐Aldrich (Dorset, UK). Paraformaldehyde was purchased from Electron Microscope Sciences (Hatfield, PA).

### Statistical Analysis

All images were analyzed offline, measuring inner diameters of arteries and staining using Imaris^®^ software (version 7.7 Bitplane). Decreases in diameter to U46619 are expressed either as % constriction (100% is equivalent to 0 μm; this established the ability of each artery to contract to U46619) or as % maximum constriction (100% is the constriction to 3 μmol/L U46619 plus 45 mmol/L KCl; used for concentration response curves to U46619). The dilation evoked by bradykinin is expressed as a reversal of the pre‐imposed 3 μmol/L U46619 tone, with 100% equivalent to the maximum inner diameter of arteries. Percentage contraction and percentage dilation are summarized as the mean±SEM, with n representing the number of arteries studied. No experiments were discarded from the study but, for most pairs of biopsies, the entire protocol could not be successfully performed on arteries from both biopsies from a patient (see Functional Studies above); therefore, means are based on denominators that are less than 12 for most outcomes. Every effort was made to pair pre‐CPB and post‐CPB data where possible; statistical power could not be improved due to completion of trial. Descriptive statistics are used to summarize unpaired data for pre‐ and post‐CPB arteries. Formal statistical comparisons on paired data first tested for Gaussian distributions (D'Agostino and Pearson omnibus normality test), which confirmed nonparametric tests should be used. For U46619 concentration response curves, individual EC_50_ values, and live cell experiments the Wilcoxon matched‐paired signed‐rank test was used (pre‐ versus post‐CPB paired biopsies).

## Results

Pre‐ and post‐CPB lung biopsies were taken from 12 patients. Patient characteristics, comorbidities, and medications are summarized in Table 1. It should be noted that all patients enrolled in this study were hypertensive, and suffered from hypercholesterolemia, and most were diabetic. For analyses based on paired biopsies when each patient served as their own control, alterations in arterial function or structure from arteries pre‐CPB to post‐CPB are at least partly attributable to the effects of the surgery and/or the use of CPB. Table 2 outlines the protocols performed on each of the arteries isolated from the 24 biopsies. Table 3 shows the summary data for arterial characteristics and the experimental protocols.

**Table 1:** Patient Characteristics

Total number of patients	12
Patient age, y	71.3±7.1
Sex (male/female)	11/1
BMI, kg/m^2^	28.3±3.7
Diabetic	58%
Previous T.I.A.	0%
Hypertensive	100%
Hypercholesterolemia	100%
Asthma	17%
Aspirin	83%
Clopidogrel	33%
Warfarin	8%
β‐Blockers	83%
Calcium channel blockers	42%
Oral nitrate	25%
Statins	92%
ACE inhibitors	58%
Angiotensin II antagonists	17%
Diuretics	8%
Insulin	17%
Oral anti‐diabetic	33%

Data presented as the mean±SD or as a percentage of the whole patient group. ACE indicates angiotensin‐converting enzyme; BMI, body mass index; T.I.A., transient ischemic attack.

**Table 2:** Protocols Performed on Arteries Isolated From Each Biopsy Obtained Pre‐ and Post‐CPB for Patients in the Study

Patient (n)	1		2		3		4		5		6		7		8		9		10		11		12	
CPB	−	+	−	+	−	+	−	+	−	+	−	+	−	+	−	+	−	+	−	+	−	+	−	+
Leak test	#	+	+	+	+	+	+	+	+	#	+	#	+	#	+	+	+	+	+	+	+	+	+	#
Contraction	−	+	+	+	+	+	+	+	+	−	+	−	+	−	+	+	+	+	+	+	+	+	+	−
Dilation	−	+	+	+	+	+	+	§	+	−	+	−	+	−	+	§	+	+	+	+	+	+	+	−
Phalloidin	−	−	+	+	+	+	+	+	+	−	−	−	−	−	−	−	−	−	+	+	+	+	−	−
Live/dead	−	−	+	+	−	−	+	+	−	−	+	−	+	−	+	+	+	+	+	+	−	−	−	−
ZO‐1	−	+	+	+	−	−	+	+	−	−	+	−	−	−	−	−	−	−	+	+	−	−	−	−

Arteries from 5 biopsies did not pass the leak test (marked #). In those arteries where contraction was not observed, the dilation could not be assessed (marked §). On 2 occasions the complete protocol was performed on arteries isolated from both pre‐ and post‐CPB biopsies (patients 2 and 10, labeled yellow and cyan, respectively, in all figures). −, protocol not performed; +, protocol performed; CPB, cardiopulmonary bypass.

**Table 3:** Summary Data Showing Functional Responses to the Vasoconstrictor U46619 and Endothelium‐Dependent Vasodilator Bradykinin in Human Pulmonary Arteries

Artery Characteristics	Pre‐CPB		Post‐CPB	
		n		n
Maximum internal diameter, μm	240±23	11	246±30	8
Number responding to vasoconstrictor (%)	11 (100)	11	6 (75)	8
Maximum constriction, μm	68±16	11	71±35	8
Percentage maximum constriction	26±5	11	27±8	8
Maximum constriction to U46619, μm	59±14	11	67±32	8
Percentage maximum constriction to U46619	23±4	11	25±10	8
Paired artery U46619 EC_50_	−7.4±1.6	7	−6.5±0.8	7
Number responding to endothelium‐dependent vasodilator (%)	5 (45)	11	2 (33)	6
Percentage maximum dilation to bradykinin	35±14	11	29±19	6
Percentage live smooth muscle cells	100±0	7	60±21	5
Percentage live endothelial cells	90±5	7	58±19	5

No significant differences were observed between pre‐ and post‐CPB. Values are the mean±SEM. 100% maximum constriction equates to a diameter of 0 μm; 100% maximum dilation equates to full reversal of U46619 tone; CPB, cardiopulmonary bypass.

### Functional Analysis

**Figure 1:**
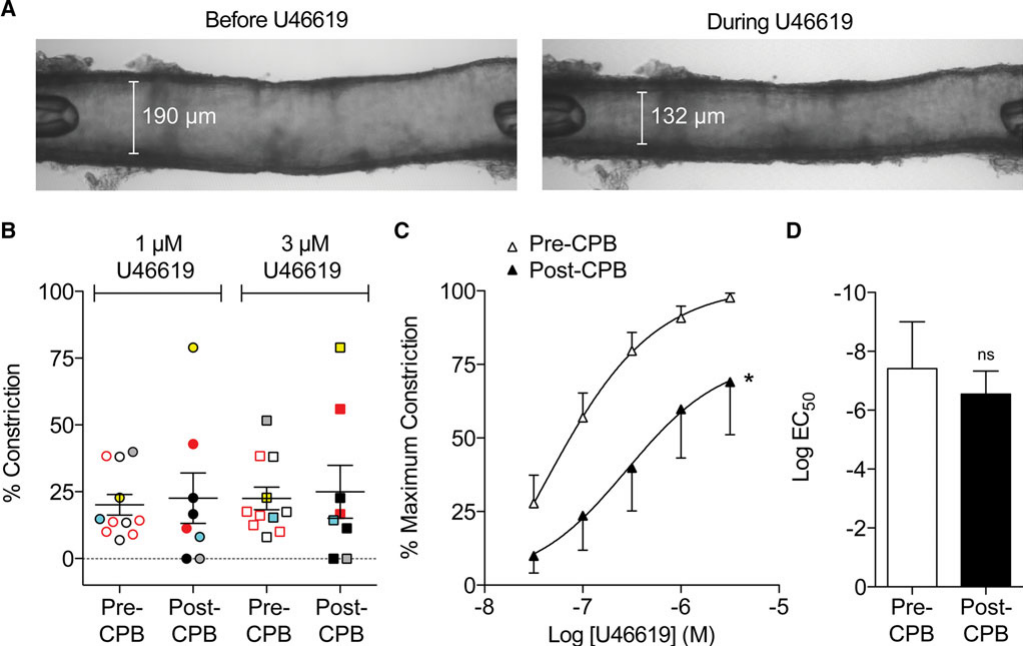
Constriction of human pulmonary arteries. A, Brightfield image showing a cannulated and pressurized human pulmonary artery before (left) and after contraction to 3 μmol/L U46619 (right). B, Contraction of human pulmonary arteries using 1 μmol/L and 3 μmol/L U44619 before (pre‐CPB, n=11) and after CPB (post‐CPB, n=8), expressed as a percentage constriction (100% equates to constriction to 0 μm). Scatter plots show individual data points for all biopsies for which measurements could be made; comparisons were made using 1‐way ANOVA with Bonferroni post hoc test, no significant difference between concentrations (1 or 3 μmol/L) or biopsy time (Pre/Post‐CPB). Yellow and blue‐filled symbols correspond to Patients 2 and 10, respectively; gray‐filled symbols indicate contractile responses from the artery shown in (A) (Patient 8); red symbols represent responses from patients taking calcium antagonist medication. C, Paired U46619 concentration response curves in human pulmonary arteries obtained from the same patients pre‐ and post‐CPB, expressed as a percentage of the maximum contraction (determined using 3 μmol/L U46619 and 45 mmol/L KCl). Values are the mean±SEM (n=7), comparison of post vs pre‐CBP arteries made using the Wilcoxon matched‐paired signed rank test (*P<0.0001 post vs pre‐CPB). D, pEC_50_ determined from (C); values are the mean±SEM (n=7) and comparisons were made using the Wilcoxon matched‐paired signed rank test; ns, not significant. CPB indicates cardiopulmonary bypass.

The average internal diameter of pre‐CPB arteries was 240±23 μm (n*=*11), which was not different from that for post‐CPB arteries 246±30 μm (n*=*8). U46619 caused at least some contraction in 100% of arteries from pre‐CPB biopsies. However, only 75% of arteries from post‐CPB biopsies contracted to U46619 (Table 3, Figure 1B). Contractile responses averaged across available pre‐ and post‐CPB arteries (unpaired) are shown in Figure 1B. When U46619 concentration response curves were compared in paired pre/post‐CPB arteries, there was a significant reduction in the % maximum constriction to U46619 (Figure 1C), without affecting the EC_50_ value (Table 3, Figure 1D).

**Figure 2:**
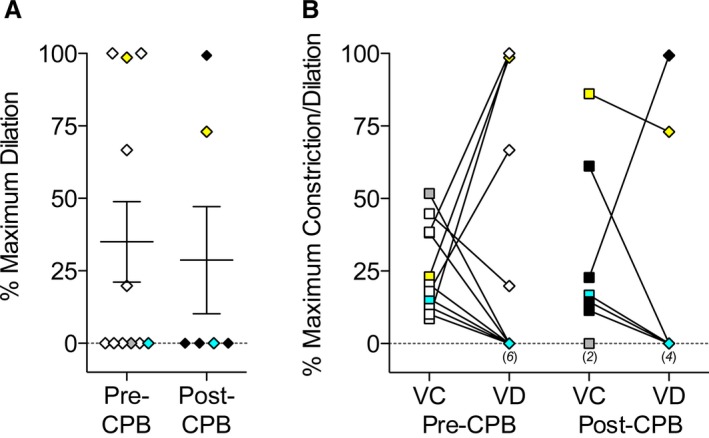
Dilation of human pulmonary arteries. A, Endothelium‐dependent vasodilation to 1 μmol/L bradykinin pre‐ (n=11) and post‐CPB (n=6). Data shown as a scatter plot showing individual responses and the mean±SEM with comparisons made using 1‐way ANOVA with Bonferroni post hoc test (not significant). B, Relationship between vasoconstriction (VC, square symbols) and vasodilation (VD, diamond symbols) responses within a given artery. Yellow, gray, and blue‐filled symbols correspond to Patients 2, 8, and 10, respectively. CPB indicates cardiopulmonary bypass; numbers in parentheses indicate the number of equal values.

Bradykinin (1 μmol/L) caused dilation in 35% of arteries from pre‐CPB biopsies, compared to 29% of arteries from post‐CPB biopsies (unpaired; Table 3, Figure 2A). The % maximum EC‐dependent dilation in pre‐CPB arteries was not different from that in post‐CPB arteries. There were no noticeable relationships between the magnitudes of vasoconstriction and vasodilation (Figure 2B).

### Structural Analysis

**Figure 3:**
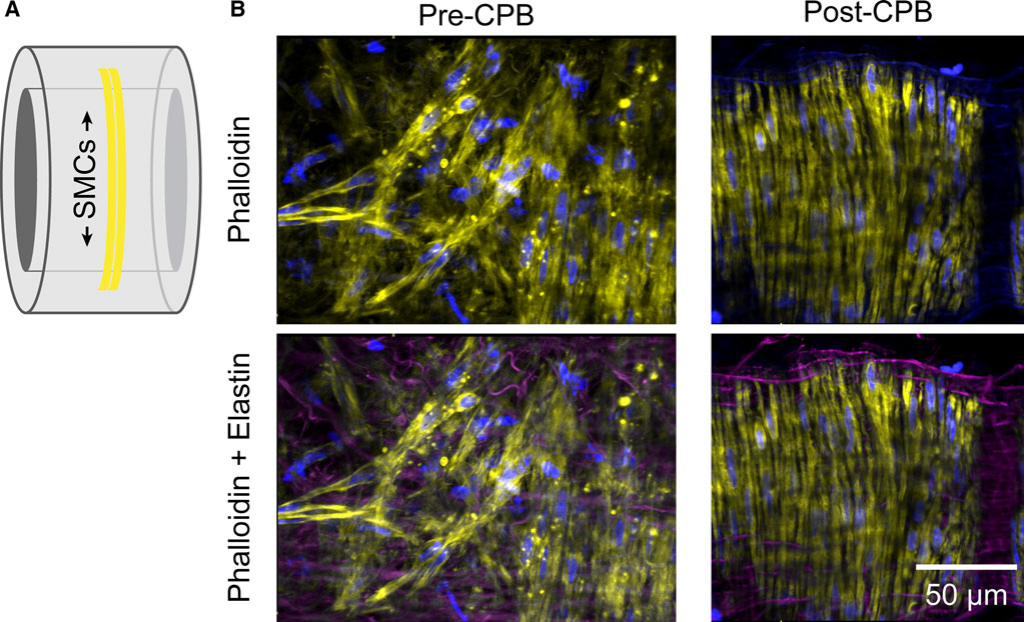
Orientation of cells in the arterial wall. A, Cartoon of arterial wall with circumferentially oriented smooth muscle cells (SMCs) vertically aligned. B, In most arteries fixed and stained with phalloidin (yellow) the SMCs were adjacent and vertically aligned, but in some cases the orientation was inconsistent and sparse. This misalignment occurred in arteries obtained both pre‐ and post‐CPB, here shown in the pre‐CPB artery; both arteries are from Patient 10. The nuclei were stained with propidium iodide (blue), and elastin with AF‐633 (pink, lower panels). CPB indicates cardiopulmonary bypass. See Video S1 for a *z*‐stack through the wall of an artery stained with AF‐633.

**Figure 4:**
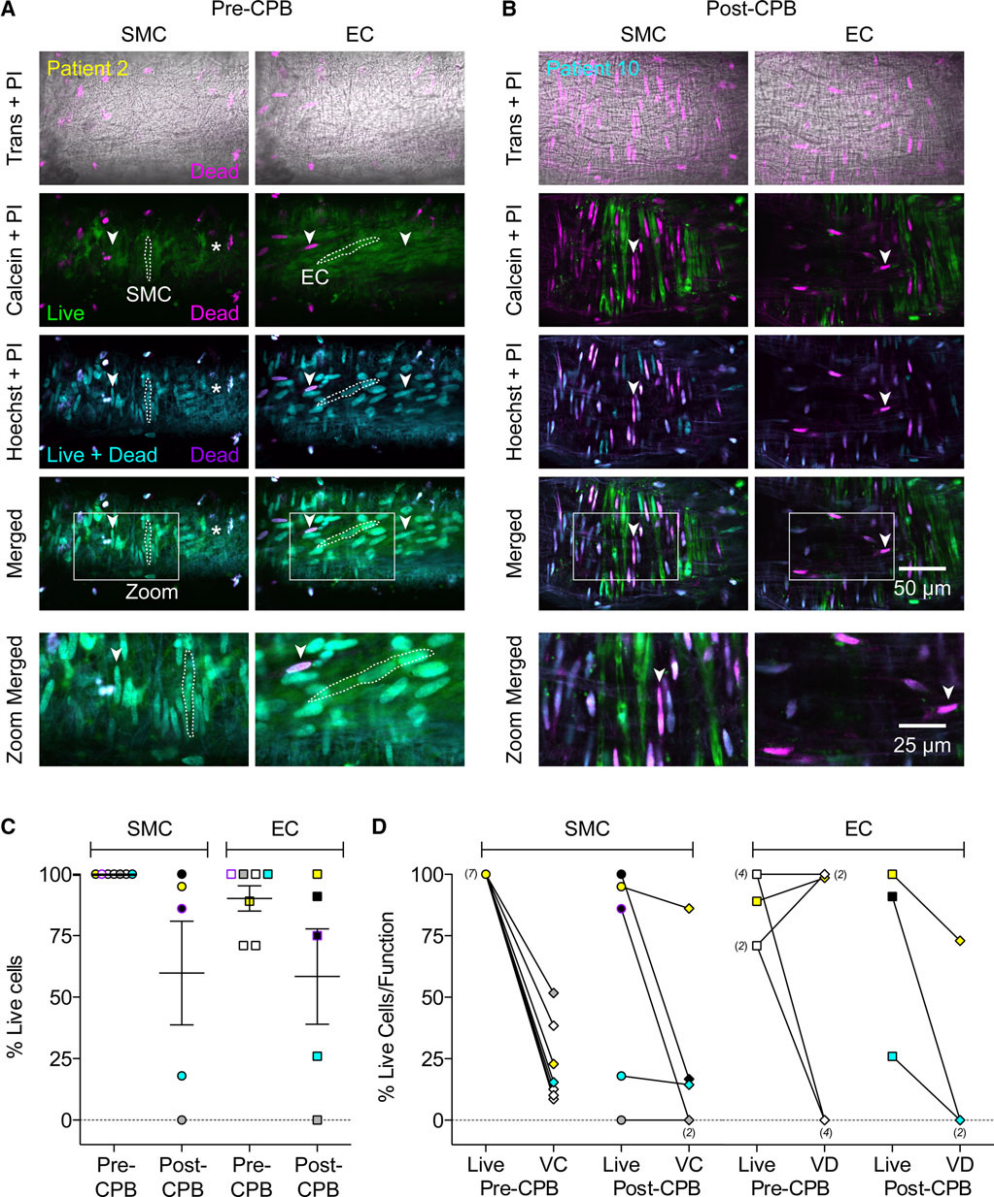
Live/dead staining in cannulated human pulmonary arteries. The cytosolic marker calcein AM is only de‐esterified in live cells (Calcein, green). The cell permeant dye Hoechst 33342 (Hoechst, cyan) stains all nuclei, whereas the cell impermeant propidium iodide (PI, pink) only penetrates dead cell membranes, co‐localized nuclear stains are purple and also reflect dead cells. Trans, transmitted light image. A, By focusing through the wall of the artery from a pre‐CPB biopsy (Patient 2), it was clear that there were no dead smooth muscle cells (SMCs, left panels, vertical orientation, one cell outlined with dashed line, no SMCs at the right of the image indicated by asterisk); and very few dead endothelial cells (ECs, right panels, near‐horizontal orientation, one cell outlined with dashed line). B, In contrast, the wall of the artery from a post‐CPB biopsy (Patient 10) was littered with dead SMCs and no live ECs were present. C, Scatter plots showing individual data points of the percentage of cells taking up calcein and not PI (Live cells) from 7 pre‐CPB biopsies, and 5 post‐CPB biopsies along with the mean±SEM. In paired comparisons there was no significant difference between pre vs post‐CPB cells (n=5, Wilcoxon matched‐paired signed‐rank test). D, Overall, the staining for live SMCs tended to reflect the ability of U46619 to stimulate vasoconstriction (VC), whereas the presence of live ECs did not uniformly reflect an ability of bradykinin to stimulate vasodilation (VD). Arrowheads indicate the same position in all images within a column. See Videos S2 and S3 for *z*‐stacks through these arteries. Yellow, gray, and cyan‐filled symbols correspond to arteries from patients 2, 8, and 10, respectively (paired data in both C and D). Purple symbols correspond to arteries from patient 4 (paired data in C). CPB indicates cardiopulmonary bypass; numbers in parenthesis indicate the number of equal values.

Staining to show the structure of fixed pulmonary artery SMC layers using DAPI (nuclear stain) and phalloidin (F‐actin stain) revealed inconsistent cell orientation and density (Figure 3). In most cases a uniform pattern was observed; however, on occasion smooth muscle structure, instead of being oriented circumferentially, was at a range of angles. This misalignment was observed in some arteries from both pre‐ and post‐CPB biopsies (Figure 3B). Furthermore, gaps were observed between SMCs (Figures 3 and 4). These gaps were often filled by elastin, confirmed by AF‐633 staining, and in some areas it was elastin that ran the entire way through the artery from the external layer through to the endothelium (Videos S1 and S2).

In arteries from pre‐CPB biopsies, live cell staining revealed that 100% of SMCs and 90% of ECs were viable (Table 3, Figure 4, Video S2). Conversely, in arteries from post‐CPB lung biopsies, only about half of the SMCs and ECs were viable (Table 3, Figure 4, Video S3). Although there was a trend for the number of live SMCs and ECs to decrease after CPB, this was not statistically significant (Table 3, n=5). When the percentage of live cells was plotted against the percentage of maximum contraction and percentage of dilation (Figure 4D), there was no positive relationship between the amount of live cells and magnitude of response.

## Discussion

This is the first study to combine pressure myography, pharmacology, and dyes to characterize both the structure and function of human pulmonary arteries. It provides proof of principle of the feasibility of carrying out such studies in the context of a clinical trial to evaluate the effects of an intervention on lung function. This is also the first study to investigate the effects of cardiac surgery and CPB on structure and function in isolated human arteries obtained from lung biopsy in patients undergoing elective CABG.

Functional studies revealed that the thromboxane mimetic U46619 caused vasoconstriction of all human pulmonary arteries isolated from pre‐CPB biopsies but not all those from post‐CPB biopsies. A paired comparison of the contractile responses of pre‐ and post‐CPB arteries revealed a significant reduction in contraction to U46619 in arteries from post‐CPB biopsies. Therefore, the inability of some arteries to contract may reflect the iatrogenic harm of cardiac surgery and CPB.

Previous structural analysis of human pulmonary arteries was last conducted in the 1970s using fixed and sectioned tissues taken from biopsies without isolating the arteries.^1^, ^2^, ^3^ This is the first study to report the structure of live and fixed isolated, cannulated, and pressurized human pulmonary arteries. The structural assessment carried out in this study shows that the interlobular human pulmonary arteries studied were partially muscular in structure, often showing incomplete coverage of the artery circumference. Furthermore, although SMC orientation was often typical (ie, wrapped around the circumference of the artery and perpendicular to flow), in some arteries this was not so, instead being oriented over a range of angles and in some places showed spiral structures similar to those seen in rat pulmonary arteries.^13^ Also, in the SMC layer there were nuclei of cells that did not appear to be SMC. Although the present study did not investigate the exact cell type, it is possible that these cells were fibroblasts or immune cells that are common components of the adventitia.^14^ It should also be noted that the gaps left in the SMC layer were often filled with AF‐633 staining, likely to represent the elastin component of the media.^15^ Altered SMC structure was found in arteries both pre‐ and post‐CPB. Therefore, in the present study we cannot link altered SMC orientation and density to CPB; rather, this may be a characteristic of this group of patients prior to surgery.

We sought to establish which of the visible SMCs were viable, so we carried out a live/dead protocol in tissues after the functional studies to investigate the possibility that decreased contraction was caused by SMC death. We showed that all SMCs were alive in arteries isolated from biopsies taken pre‐CPB, whereas only half the SMCs were alive in arteries from biopsies taken post‐CPB. One explanation for these findings is that CPB causes decreased arterial contraction due to damage to SMCs in the arterial wall. During CPB, there is minimal blood perfusion of pulmonary arteries/arteries and decreased oxygenation; these conditions are known to provoke an increased immunological response through ischemia–reperfusion injury and mechanical stress, causing atelectasis and lung dysfunction.^16^, ^17^, ^18^ Indeed, a recent study has shown that CPB carried out in rabbits caused an increase in leakage of Evans blue dye administered intravenously, which could be inhibited by an antibody against tumor necrosis factor α.^19^ Therefore, macrophage and neutrophils released during CPB could cause tumor necrosis factor α–induced apoptosis in pulmonary SMCs, such as that seen in this study.

Functional studies of ECs suggested decreased endothelium‐dependent vasodilation after CPB. In arteries from pre‐CPB biopsies, less than half were capable of endothelium‐dependent vasodilation, with an average dilation <50%. However, in arteries from biopsies taken after CPB, dilation to bradykinin was measurable in only 1 of 5. As with the contraction findings, this is consistent with a previous finding in porcine coronary arterioles of a slight decrease in vasorelaxation after CPB, which was associated with increased EC adhesion molecules and myeloperoxidase leading to cell death.^20^ Our structural studies, which showed a smaller percentage of live cells in arteries from post‐CPB biopsies compared to pre‐CPB biopsies, further support this hypothesis.

EC orientation, as for SMC, was atypical. Irrespective of the timing of biopsies in relation to CPB, ECs were often arranged at various angles instead of running in line with blood flow. This finding has previously been associated with altered shear stress associated with some vascular disease.^21^, ^22^

In conclusion, we have demonstrated the feasibility of obtaining and studying the function and structure of arteries from human lung biopsies. We have shown that CPB is detrimental to both SMC and EC function in pulmonary arteries and that this is potentially a consequence of cell death. The ability to distinguish changes that are likely to be attributable to CPB suggests that these methods could be used to evaluate the effects of interventions to reduce the adverse consequences of CPB on the lungs.

## Sources of Funding

This work was generously supported by the British Heart Foundation (BHF; FS/13/16/30199, PG/13/9/29990), and the Oxford BHF Centre of Research Excellence; the Garfield Weston Trust; and the UK National Institute for Health Research (NIHR) Bristol Cardiovascular Biomedical Research Unit. Kim Dora is a BHF‐funded Senior Basic Science Research Fellow; Al Jaaly is a NIHR‐funded Academic Clinical Fellow.

## Disclosures

None.

## Supporting information


**Video S1.** Structure of human pulmonary arteries. Confocal *z*‐stack through the wall of a pressurized artery showing nuclei (blue), elastin (pink), and tight junctions (green). Note that interendothelial cell tight junctions are present both in the lumen of the artery, and on the outside. The luminal surface does not appear uniformly populated with endothelial cells. Artery obtained post–cardiopulmonary bypass.

**Videos S2 and S3.** Pre‐ and post–cardiopulmonary bypass (CPB) artery live‐dead cell stains. Simultaneous confocal *z*‐stacks through the walls of 2 pressurized arteries (Pre‐CPB from Patient 2, Video S2; Post‐CPB from Patient 10, Video S3) showing live cells with calcein (green), all nuclei with Hoechst 33342 (cyan), and dead cells with propidium iodide (pink) often co‐localized with Hoechst 33342 (purple). Note the background fluorescence of elastin is also visible. See Figure 4 for details. Scale bars, 30 μm.

## References

[1] Townsley MI . Structure and composition of pulmonary arteries, capillaries, and veins. Compr Physiol. 2012;2:675–709. 23606929 10.1002/cphy.c100081 PMC3630377

[2] Kubo K , Ge RL , Koizumi T , Fujimoto K , Yamanda T , Haniuda M , Honda T . Pulmonary artery remodeling modifies pulmonary hypertension during exercise in severe emphysema. Respir Physiol. 2000;120:71–79. 10786646 10.1016/s0034-5687(00)00090-6

[3] Aiello VD , Gutierrez PS , Chaves MJ , Lopes AA , Higuchi ML , Ramires JA . Morphology of the internal elastic lamina in arteries from pulmonary hypertensive patients: a confocal laser microscopy study. Mod Pathol. 2003;16:411–416. 12748246 10.1097/01.MP.0000067685.57858.D7

[4] Taggart DP , el‐Fiky M , Carter R , Bowman A , Wheatley DJ . Respiratory dysfunction after uncomplicated cardiopulmonary bypass. Ann Thorac Surg. 1993;56:1123–1128. 8239811 10.1016/0003-4975(95)90029-2

[5] Gao D , Grunwald GK , Rumsfeld JS , Mackenzie T , Grover FL , Perlin JB , McDonald GO , Shroyer AL . Variation in mortality risk factors with time after coronary artery bypass graft operation. Ann Thorac Surg. 2003;75:74–81. 12537196 10.1016/s0003-4975(02)04163-2

[6] Ascione R , Lloyd CT , Underwood MJ , Lotto AA , Pitsis AA , Angelini GD . Inflammatory response after coronary revascularization with or without cardiopulmonary bypass. Ann Thorac Surg. 2000;69:1198–1204. 10800819 10.1016/s0003-4975(00)01152-8

[7] Rothenburger M , Soeparwata R , Deng MC , Schmid C , Berendes E , Tjan TD , Wilhelm MJ , Erren M , Bocker D , Scheld HH . Prediction of clinical outcome after cardiac surgery: the role of cytokines, endotoxin, and anti‐endotoxin core antibodies. Shock. 2001;16(suppl 1):44–50. 11770033 10.1097/00024382-200116001-00009

[8] Ascione R , Lloyd CT , Underwood MJ , Lotto AA , Pitsis AA , Angelini GD . Economic outcome of off‐pump coronary artery bypass surgery: a prospective randomized study. Ann Thorac Surg. 1999;68:2237–2242. 10617009 10.1016/s0003-4975(99)01123-6

[9] Johnson D , Thomson D , Hurst T , Prasad K , Wilson T , Murphy F , Saxena A , Mayers I . Neutrophil‐mediated acute lung injury after extracorporeal perfusion. J Thorac Cardiovasc Surg. 1994;107:1193–1202. 8176961

[10] Schreiber JU , Lance MD , de Korte M , Artmann T , Aleksic I , Kranke P . The effect of different lung‐protective strategies in patients during cardiopulmonary bypass: a meta‐analysis and semiquantitative review of randomized trials. J Cardiothorac Vasc Anesth. 2012;26:448–454. 22459933 10.1053/j.jvca.2012.01.034

[11] Fiorentino F , Angelini GD , Suleiman MS , Rahman A , Anderson J , Bryan AJ , Culliford LA , Moscarelli M , Punjabi PP , Reeves BC . Investigating the effect of remote ischaemic preconditioning on biomarkers of stress and injury‐related signalling in patients having isolated coronary artery bypass grafting or aortic valve replacement using cardiopulmonary bypass: study protocol for a randomized controlled trial. Trials. 2015;16:181. 25899533 10.1186/s13063-015-0696-z PMC4425928

[12] Bagher P , Beleznai T , Kansui Y , Mitchell R , Garland CJ , Dora KA . Low intravascular pressure activates endothelial cell TRPV4 channels, local Ca^2+^ events, and IK_Ca_ channels, reducing arteriolar tone. Proc Natl Acad Sci USA. 2012;109:18174–18179. 23071308 10.1073/pnas.1211946109 PMC3497745

[13] Ortiz PP , Diaz P , Daniel‐Lamaziere JM , Lavallee J , Bonnet J , Torres A , Whyte J , Bernal J , Sarrat R . Morphometry of the human splenic artery: muscular columns, morphofunctional aspects and developmental implications. Histol Histopathol. 1998;13:315–324. 9589889 10.14670/HH-13.315

[14] Stenmark KR , Nozik‐Grayck E , Gerasimovskaya E , Anwar A , Li M , Riddle S , Frid M . The adventitia: essential role in pulmonary vascular remodeling. Compr Physiol. 2011;1:141–161. 23737168 10.1002/cphy.c090017 PMC4169049

[15] Clifford PS , Ella SR , Stupica AJ , Nourian Z , Li M , Martinez‐Lemus LA , Dora KA , Yang Y , Davis MJ , Pohl U , Meininger GA , Hill MA . Spatial distribution and mechanical function of elastin in resistance arteries: a role in bearing longitudinal stress. Arterioscler Thromb Vasc Biol. 2011;31:2889–2896. 21979438 10.1161/ATVBAHA.111.236570 PMC3380608

[16] Ng CS , Wan S , Yim AP , Arifi AA . Pulmonary dysfunction after cardiac surgery. Chest. 2002;121:1269–1277. 11948063 10.1378/chest.121.4.1269

[17] Pinhu L , Whitehead T , Evans T , Griffiths M . Ventilator‐associated lung injury. Lancet. 2003;361:332–340. 12559881 10.1016/S0140-6736(03)12329-X

[18] Goebel U , Siepe M , Mecklenburg A , Doenst T , Beyersdorf F , Loop T , Schlensak C . Reduced pulmonary inflammatory response during cardiopulmonary bypass: effects of combined pulmonary perfusion and carbon monoxide inhalation. Eur J Cardiothorac Surg. 2008;34:1165–1172. 18829339 10.1016/j.ejcts.2008.07.031

[19] Yu Y , Gao M , Li H , Zhang F , Gu C . Pulmonary artery perfusion with anti‐tumor necrosis factor alpha antibody reduces cardiopulmonary bypass‐induced inflammatory lung injury in a rabbit model. PLoS One. 2013;8:e83236. 24386164 10.1371/journal.pone.0083236 PMC3873915

[20] Serraf A , Sellak H , Herve P , Bonnet N , Robotin M , Detruit H , Baudet B , Mazmanian GM , Planche C . Vascular endothelium viability and function after total cardiopulmonary bypass in neonatal piglets. Am J Respir Crit Care Med. 1999;159:544–551. 9927371 10.1164/ajrccm.159.2.9803024

[21] Mazzolai L , Bouzourene K , Hayoz D , Dignat‐George F , Liu JW , Bounameaux H , Dunoyer‐Geindre S , Kruithof EK . Characterization of human late outgrowth endothelial progenitor‐derived cells under various flow conditions. J Vasc Res. 2011;48:443–451. 21625177 10.1159/000324844

[22] DeMaio L , Chang YS , Gardner TW , Tarbell JM , Antonetti DA . Shear stress regulates occludin content and phosphorylation. Am J Physiol Heart Circ Physiol. 2001;281:H105–H113. 11406474 10.1152/ajpheart.2001.281.1.H105

